# Uniportal Versus Multiportal Video-Assisted Thoracoscopic Lobectomy for Lung Cancer: An Updated Meta-analysis

**DOI:** 10.1007/s00408-020-00411-9

**Published:** 2021-01-02

**Authors:** Dimitrios E. Magouliotis, Maria P. Fergadi, Kyriakos Spiliopoulos, Kalliopi Athanassiadi

**Affiliations:** 1 Division of Surgery and Interventional Science, Faculty of Medical Sciences, UCL, London, UK; 2 Department of Surgery, University of Thessaly, Biopolis, 41110 Larissa, Greece; 3 Unit of Thoracic Surgery, Evangelismos Hospital, Athens, Greece; 4 Faculty of Medicine, University of Thessaly, Biopolis, Larissa, Greece; 5 Department of Thoracic and Cardiovascular Surgery, Larissa University Hospital, Larissa, Greece

**Keywords:** Video-assisted thoracoscopic surgery, Vats, Uniportal, Uvats, Single incision, Lung cancer

## Abstract

**Objective:**

We reviewed the available literature on patients with lung cancer undergoing either uniportal (UVATS) or multiport video-assisted thoracoscopic surgery (MVATS).

**Methods:**

Original research studies that evaluated perioperative and long-term outcomes of UVATS versus MVATS were identified, from January 1990 to April 2020. The perioperative, along with the oncologic and long-term survival outcomes, were calculated according to either a fixed or a random effect model, appropriately. The *Q* statistics and *I*^*2*^ statistic were used to test for heterogeneity among the studies.

**Results:**

Twenty studies were included, incorporating a total of 1,469 patients treated with UVATS and 3,231 treated with MVATS. The incidence of complications was lower in patients treated with UVATS [OR: 0.76 (95% CI 0.62, 0.93); *p* = 0.008]. The chest tube duration was significantly lower in the UVATS group (WMD: − 0.63 [95% CI − 1.03, − 0.23]; p = 0.002). Length of hospital stay (L.O.S.) was also lower in the UVATS patient group (WMD: − 0.54 [− 0.94, − 0.13]; p = 0.009), along with postoperative pain [WMD: − 0.57 (95% CI − 0.97, − 0.18); *p* = 0.004]. No significant differences were found regarding the mean operative time (M.O.T.), mean blood loss, the number of resected lymph nodes, the 30-day mortality, along with the survival at 1 and 3 years postoperatively.

**Conclusions:**

The present meta-analysis indicates that UVATS is associated with enhanced outcomes in patients undergoing surgery for lung cancer. Well-designed, randomized studies, comparing UVATS to MVATS, are necessary to further assess their long-term clinical outcomes.

**Supplementary Information:**

The online version contains supplementary material available at 10.1007/s00408-020-00411-9.

## Introduction

Minimally invasive surgical techniques have become the standard approach for treating patients with non-small cell lung cancer [1]. In this context, the conventional multiport video-assisted thoracoscopic surgery (MVATS) is generally performed through two to four incisions, thus, allowing multiple different angles of approach to the hilar structures and lymphatic tissues during thoracoscopic lobectomy [2]. With increased experience and the development of enhanced surgical instruments for VATS, thoracoscopic techniques continue to improve, by decreasing the working port size and the number of incisions, thus, becoming the mainstream in most centers [3].

Recently, the uniportal VATS (UVATS) approach has been proposed as a feasible alternative to the multiport VATS to perform feasibly and safely a wide range of thoracic surgical operations, including diagnostic procedures, minor and major lung anatomical resections, along with the excision of mediastinal tumors [4, 5]. UVATS has been reported to have advantages including less postoperative pain, less paresthesia, and with better patient satisfaction. However, certain concerns have been raised regarding the safety and feasibility of UVATS due to its technical difficulties. Recently, a Delphi consensus report from the Uniportal VATS Interest Group (UVIG) of the European Society of Thoracic Surgeons (ESTS) was published. The report concluded that UVATS is a valid alternative to MVATS [6]. Nonetheless, the same report called for newer studies with longer follow-up and randomized design to fully evaluate whether it should be performed in selected cases/centers [6]. As the number of studies comparing the feasibility and safety of UVATS and MVATS increases, and given the take-home messages of the ESTS consensus report [6], it is necessary to reevaluate whether the results between the two techniques are at least equivalent. The purpose of the present study was to summarize and analyze the existing data by comparing the surgical outcomes of UVATS and MVATS, in order to provide the best evidence that is currently available.

## Materials and Methods

### Search Strategy and Articles Selection

The present study was conducted following the protocol agreed by all authors and according to the Preferred Reporting Items for Systematic Reviews and Meta-Analyses (PRISMA) guidelines [7]. No institutional approval or recent consent was needed for the present study. The study protocol was registered in Research Registry database (unique identifying number: researchregistry6184). The PICO (population, intervention, control, and outcome) criteria were used to form the research question, as demonstrated in Table S1. A literature search was performed in four databases: (i) Pubmed (Medline), (ii) Cochrane Central Register of Controlled Studies (CENTRAL), (iii) EMBASE, and (iv) Scopus (ELSEVIER) (last search: September 10^th^, 2020) using the following terms in every possible combination: “uniportal,” “single-incision,” “single-port,” “uvats,” “video-assisted thoracoscopic surgery,” “vats,” “lobectomy,” “lung cancer,” and “non-small cell lung cancer.” Inclusion criteria were (1) original comparative reports with ≥ 10 patients, (2) written in the English language, (3) published from 1990 to 2020, (4) conducted on human subjects, and (5) reporting outcomes of patients undergoing UVATS and MVATS lobectomy for lung cancer. Studies reporting outcomes on sublobar resections and duplicate articles were excluded, where multiple studies analyzed the same population only the larger study or the one with the longest follow-up was included in the qualitative and quantitative analysis. The reference lists of all included articles were also reviewed for additional studies. Two independent reviewers (DEM, MPF) extracted the data from the included studies. Both reviewers have received certified education regarding systematic literature search. Any discrepancies between the investigators about the inclusion or exclusion of studies were discussed with the senior author (KA) in order to include articles that best matched the criteria until consensus was reached. The authors had personal equipoise concerning the best intervention. The kappa coefficient test was applied in order to assess the level of agreement between the authors regarding the inclusion and exclusion of studies.

### Data Extraction

For each eligible study, data were extracted relative to demographics (number of patients, sex, mean age, histology, stage of disease), type of VATS approach (UVATS or MVATS) according to UVIG-ESTS criteria [6], perioperative parameters, and long-term survival. The perioperative short-term outcomes were the primary endpoints, and the long-term survival was the secondary endpoint. Besides, categorical outcomes were 2 × 2 tabulated, referring patients presenting the outcome and patients free of the outcome, separately for UVATS and MVATS groups. Regarding continuous outcomes, we extracted the mean, the standard deviation (SD), and the number of patients. In cases that SD was not available, it was calculated using the available data.

### Statistical Analysis

The results were analyzed using RevMan 5.3® (The Cochrane Collaboration, St Albans House, 57–59 Haymarket, London SW1Y 4QX, United Kingdom) and in accordance with the guidelines for the European Journal of Cardio-Thoracic Surgery and the Interactive Cardiovascular and Thoracic Surgery [8]. Regarding the categorical outcomes, the Odds Ratio (ORs) and 95% confidence interval (95% CI) were calculated, based on the extracted data, employing random effects (Mantel–Haenszel statistical method). OR < 1 denoted outcome was more frequent in the MVATS group. Continuous outcomes were evaluated by means of weighted mean difference (WMD) with its 95% CI, using random-effects (Inverse Variance statistical method) models to calculate pooled effect estimates. In cases where WMD < 0, values in the MVATS group were higher. We selected the random-effects model since we did not expect that all the included studies would share a common effect size. Inter-study heterogeneity was assessed through Cochran *Q* statistic and by estimating *I*^2^ [9]. High heterogeneity renders the outcome less valid. A *p* value of less than 0.05 was set as the threshold indicating a statistically significant result.

### Quality and Publication Bias Assessment

The Newcastle–Ottawa Quality Assessment Scale (NOS) [10] was used as an assessment tool to evaluate non-RCTs. The scale’s range varies from zero to nine stars, and studies with a score equal to or higher than five were considered to have the adequate methodological quality to be included. The RCTs were assessed for their methodological quality with the tools used to evaluate the risk of bias according to the Cochrane Handbook for Systematic Reviews of Interventions [9]. Two reviewers (DEM and MPF) rated the studies independently, and a final decision was reached by consensus. Visual inspection of funnel plot asymmetry was performed to address possible small-study effects.

## Results

### Article Selection and Patient Demographics

**Fig. 1:**
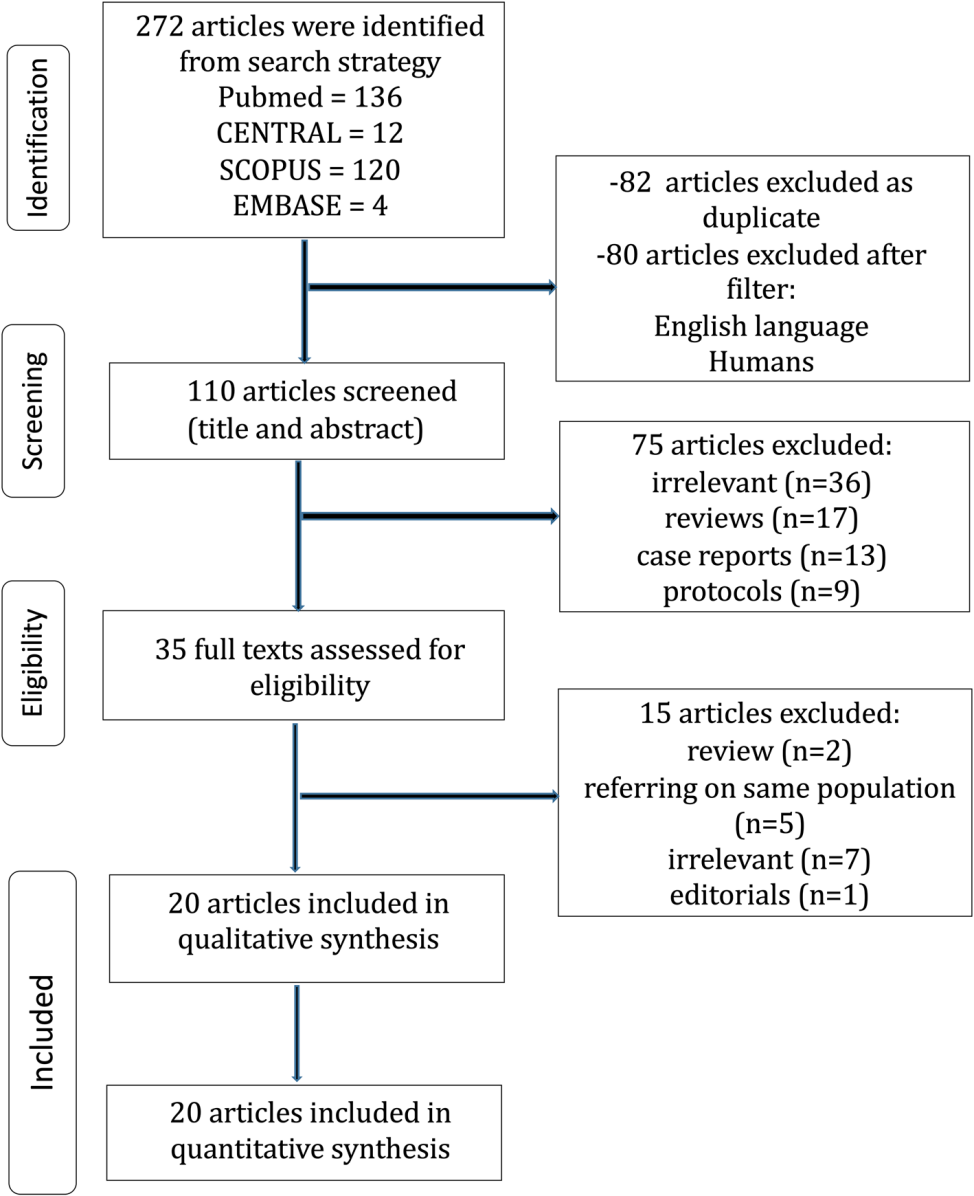
UVATS versus MVATS for lung cancer flow diagram

**Fig. 2:**
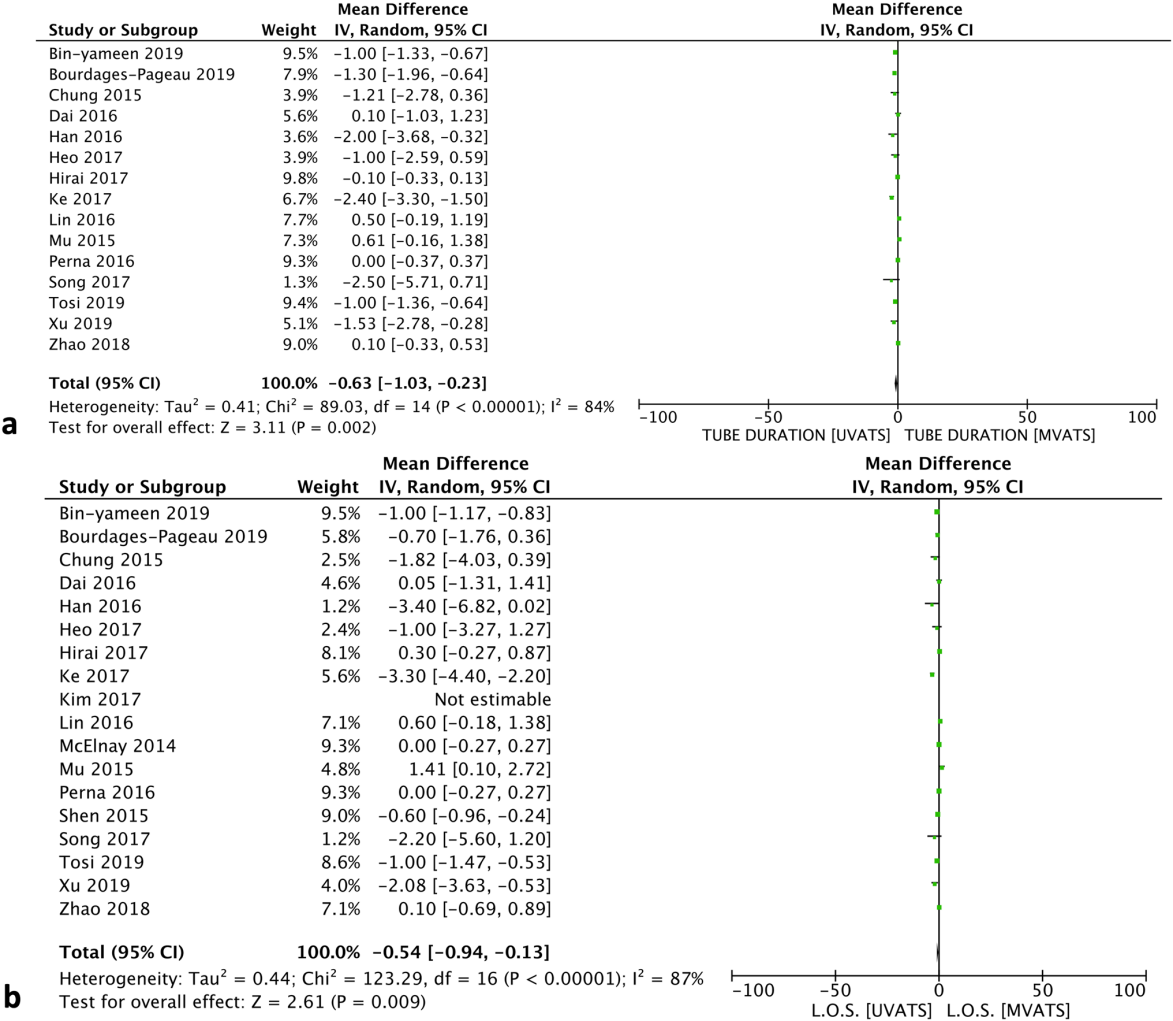
Forest plots describing the differences in **a** chest tube duration, **b** length of stay (L.O.S.). **a** Chest tube duration was shorter in the UVATS group. **b** L.O.S. was shorter in the UVATS group. *IV* inverse variance statistical method; *95% CI* 95% Confidence intervals

**Fig. 3:**
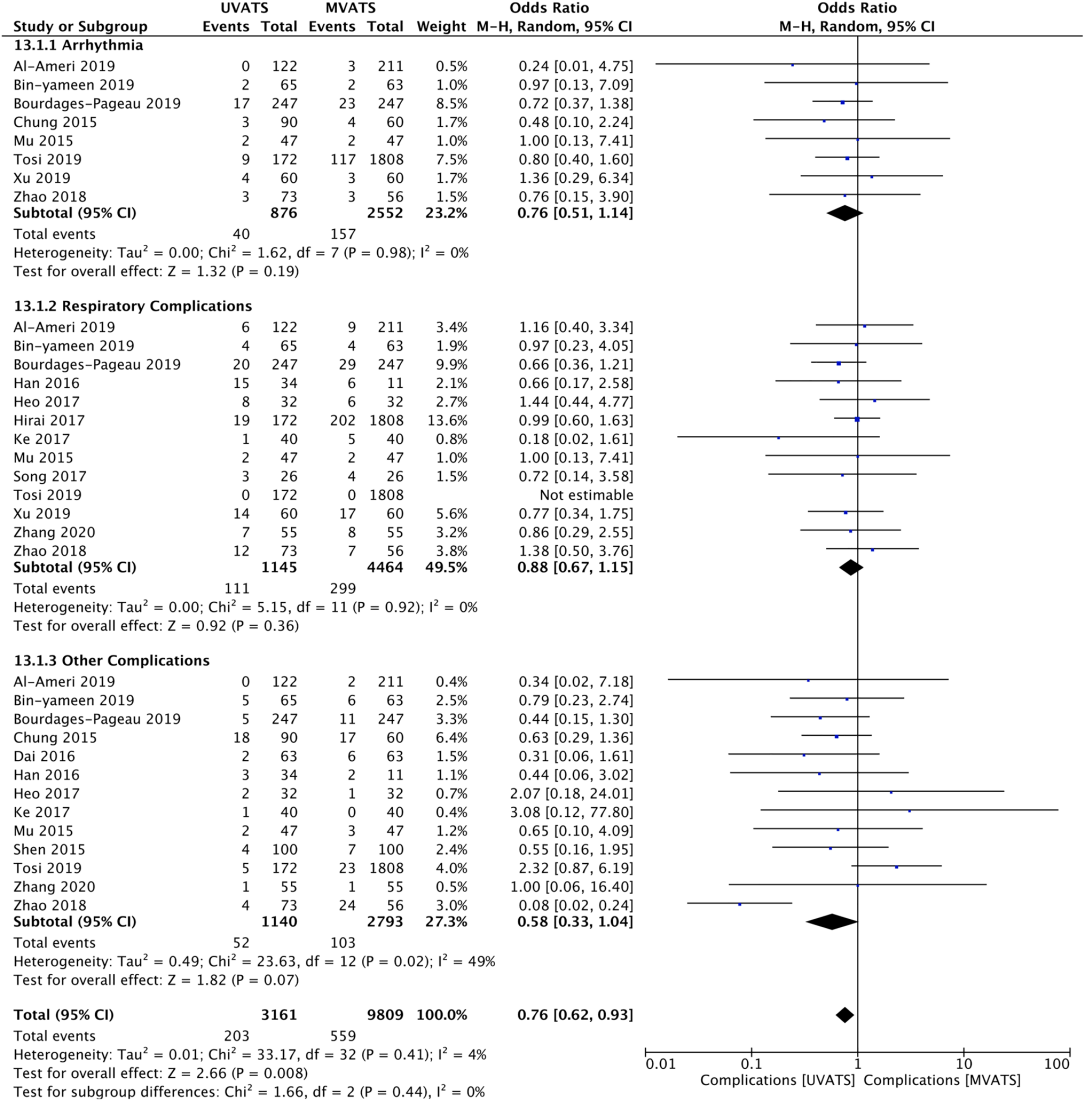
Forest plot describing the differences in total complications, along with subgroup analysis regarding arrhythmias and respiratory complications. Total complications were fewer in the UVATS group. No significant difference was found regarding the rate of arrhythmias and respiratory complications between the two groups. *M-H* Mantel–Haenszel statistical method; *95% CI* 95% Confidence Interval

The flow diagram of the search of the literature is shown in Fig. 1 and the Prisma Checklist is provided as supplementary material. The baseline characteristics of the included studies are summarized in Table 1. Among the 272 articles in Pubmed, Scopus, EMBASE, and CENTRAL that were retrieved, twenty studies were included in the qualitative and quantitative synthesis [10–30]. The two reviewers reached a “substantial” level of agreement regarding the studies that were finally included (kappa = 0.787; 95% CI 0.659, 0.914). The study design was randomized controlled in two studies [24, 29], prospective in three studies [22, 23, 28], and retrospective in fifteen studies [11–21, 25–27, 30]. The included studies were conducted in Sweden [11], Canada [12, 13], South Korea [14, 16, 17, 20, 26], China [15, 19, 21, 23, 25, 28, 30], Japan [18], UK [22], Spain [24], Italy [27], and Pakistan [29] and were published between 2014 and 2020. The UVATS and MVATS sample size ranged from 15 to 172 and from 11 to 1808 patients, respectively. The total sample size was 4,700 patients: 1,469 patients treated with UVATS and 3,231 patients treated with MVATS. According to the UVIG-ESTS consensus report [6], eligibility for UVATS lobectomy should include tumors with T1/T2 and N0/N1 status. The report also recommended incision length ≤ 4 cm and systematic dissection of all of the ipsilateral lymph nodes, while chest wall involvement was not considered an absolute contraindication. In fact, the majority of the patients included in the present meta-analysis presented T1/T2, N0/N1 status, as demonstrated in Table S3, while the incision length and the systematic dissection of all ipsilateral lymph nodes were in accordance with the UVIG-ESTS criteria in all studies. The baseline characteristics of studies comparing the outcomes between patients treated with either UVATS or MVATS are provided in Tables 1, S2, S3, and the pooled estimates in Table 2, Figs. 2, 3, S1. The Newcastle–Ottawa rating scale assessment for all studies is shown in Table 1.

**Table 1:** Characteristics of the studies that were finally included in the meta-analysis

Study ID, Year	Journal	Country	Time Period	Type of Study	Patients, n		Females, n (%)		Mean Age, years (SD)		BMI, kg/m^2^, mean (SD)		ASA Score III/IV		FEV_1_, %		NOS
					UVATS	MVATS	UVATS	MVATS	UVATS	MVATS	UVATS	MVATS	UVATS	MVATS	UVATS	MVATS	
Al-Ameri et al. 2019 [11]	J Thorac Dis	Sweden	2016–2018	R	122	211	74 (60.7)	139 (65.9)	67.5 (9.0)	67.7 (8.2)	25.3 (4.2)	25.6 (4.5)	Ν/Α	Ν/Α	2.39 (0.8)	2.30 (0.61)	6
Bin Yameen et al. 2019 [12]	Can J Surg	Canada	2012–2016	R	65	63	33 (51)	42 (67)	67.6 (8.9)	69.2 (7.9)	29.0 (7.8)	27.0 (4.5)	N/A	N/A	2.0 (0.6)	2.0 (0.6)	6
Bourdages –Pageau et al. 2019 [13]	Semin ThoracicSurg	Canada	2014–2017	R	247	247	153 (62)	158 (64)	65.7 (7.9)	65.6 (7.8)	27.1 (5.5)	26.9 (5.4)	2.7 (0.8)	2.7 (0.8)	88.1* (16.1)	88.2 (19.6)	7
Chung et al. 2015 [14]	Interact CardioVasc Thorac Surg	South Korea	2013- 2014	R	90	60	40 (44)	26 (43.3)	60.54 (11.38)	63.30 (9.29)	N/A	N/A	N/A	N/A	N/A	N/A	6
Dai et al. 2016 [15]	J Thorac Dis	China	2013–2015	R	63	63	23 (36.51)	17 (26.98)	58.68 (9.24)	57.11 (12.22)	21.99 (3.06)	22.04 (3.01)	N/A	N/A	1.99 (0.40)	1.98 (0.38)	7
Han et al. 2016 [16]	J Thorac Dis	South Korea	2006–2015	R	34	11	12 (26.7)	60 (13)	N/A	N/A	N/A	6					
Heo et al. 2017 [17]	Korean J Thorac CardioVasc Surg	South Korea	2012–2015	R	32	32	10 (31.3)	13 (40.6)	66 (40–77)	64 (46–82)	23.5 (17.7–31.7)	24.6 (15.5–33.7)	N/A	N/A	88.0* (66.0–131.0)	87.5 (65.0–123.0)	6
Hirai et al. 2017 [18]	Eur J Cardio-thorac Surg	Japan	2008–2015	R	80	80	N/A	N/A	N/A	N/A	N/A	N/A	N/A	N/A	1.88 (0.32)	1.65 (0.41)	6
Ke et al. 2017 [19]	J Thorac Dis	China	2014–2016	R	40	40	23 (57.5)	22 (55.0)	59.3 (11.2)	60.2 (11.7)	19.5 (2.3)	20.7 (2.0)	N/A	N/A	75.4 * (11.6)	77.6* (12.1)	7
Kim et al. 2017 [20]	J Thorac Oncol	South Korea	2006–2015	R	76	76	N/A	N/A	N/A	N/A	N/A	N/A	N/A	N/A	N/A	N/A	6
Lin et al. 2016 [21]	Pak J Med Sci	China	2013–2014	R	21	46	8 (38.09)	17 (36.95)	59 (7.3)	62 (6.2)	N/A	N/A	N/A	N/A	N/A	N/A	6
McElnay et al. 2014 [22]	Eur Cardio- Thorac Surg	UK	2012–2013	P	15	95	7 (47)	46 (48)	70 (66–77)	69 (61–74)	24 (22.5–28)	26 (24–29)	4 (27)	17 (18)	N/A	N/A	7
Mu et al. 2015 [23]	Chin Med J	China	2014–2015	P	47	47	22 (46.8)	33 (70.2)	56.67 (11.62)	60.77 (11.04)	N/A	N/A	N/A	N/A	2.39 (0.60)	2.41 (0.59)	7
Perna et al. 2016 [24]	Eur J Cardio-Thorac Surg	Spain	2013–2015	RCT	51	55	16 (31.4)	20 (36.4)	69 (65–76)	72 (68–79)	N/A	N/A	2 (3.9)	1 (1.8)	N/A	N/A	–
Shen et al. 2015 [25]	Eur J Cardio-Thorac Surg	China	2013–2014	R	100	100	44 (44)	45 (45)	61.5 (7.9)	60.9 (7.8)	N/A	N/A	N/A	N/A	2.32 (0.31)	2.31 (0.63)	7
Song et al. 2017 [26]	Korean J ThoracCardiovasc Surg	South Korea	2011–2016	R	26	26	11 (42.3)	11 (42.3)	64.8 (9.7)	65.0 (9.4)	N/A	N/A	N/A	N/A	2.3 (0.7)	2.3 (0.4)	6
Tosi et al. 2019 [27]	Interact CardioVasc Thorac Surg	Italy	2014–2017	R	172	1808	83 (40.3)	819 (45.3)	69.5 (11.3)	68.0 (12.0)	N/A	N/A	N/A	N/A	92.0* (25.9)	96.0* (27)	7
Xu et al. 2019 [28]	Thoracic Cancer	China	2017	P	60	60	27 (45)	29 (48.3)	61.35 (10.76)	63.50 (9.64)	21.70 (2.24)	22.38 (2.88)	N/A	N/A	N/A	N/A	7
Zhang et al. 2020 [29]	Pak J Med Sci	Pakistan	2017–2018	RCT	55	55	27 (49.09)	26 (47.27)	61.3 (1.4)	61.3 (1.2)	N/A	N/A	N/A	N/A	1.60 (0.53)	1.02 (0.15)	–
Zhao et al. 2019 [30]	Med J	China	2013–2015	R	73	56	14 (19.17)	9 (16.07)	67.5 (4.6)	67.3 (5.3)	22.2 (2.5)	21.8 (2.6)	N/A	N/A	2.0 (0.4)	2.0 (0.4)	6

The first author of every study along with the year of publication, the country of origin, the study design, the type of P/D according to IASLC-ISC criteria, the number of participants, the number of female patients, along with the mean age and the number of stars according to the Newcastle–Ottawa Quality Assessment Scale (NOS)

The Newcastle–Ottawa Scale (NOS) for assessing the quality of non-randomized studies. Every study is judged on three perspectives: the selection, the comparability, and the ascertainment of the exposure of the study groups. The highest quality studies are awarded up to 9 stars

*RCT* randomized controlled trial; *R* retrospective; *P* prospective; *N/A* not available; *UVATS* uniportal video-assisted thoracoscopic surgery; *MVATS* multiportal video-assisted thoracoscopic surgery, *NOS* Newcastle–Ottawa Scale; *SD* standard deviation

**Table 2:** Summary of the analysis of the categorical and continuous outcomes

Categorical outcomes	*n*	OR (95% CI)	*p*	Heterogeneity	
				*I*^*2*^	*p*
Total complications	35	0.76 [0.62, 0.93]	0.008	4%	0.41
Arrhythmias	8	0.76 [0.51, 1.14]	0.19	0%	0.98
Respiratory complications	13	0.88 [0.67, 1.15]	0.36	0%	0.92
Conversions	11	0.91 [0.56, 1.46]	0.68	0%	0.49
30-day mortality	12	0.51 [0.10, 2.47]	0.40	0%	0.62
1-year survival	2	0.80 [0.24, 2.68]	0.72	0%	0.70
3-year survival	3	0.73 [0.25, 2.11]	0.56	70%	0.04

Continuous outcomes	*n*	WMD (95% CI)		*I*^*2*^	*p*
FEV1	9	0.11 [− 0.03, 0.25]	0.12	85%	< 0.01
Age	17	− 0.43 [− 1.23, 0.37]	0.29	62%	< 0.01
Tumor size	11	0.10 [− 0.07, 0.27]	0.26	70%	< 0.01
M.O.T	18	9.37 [− 0.66, 19.40]	0.07	93%	< 0.01
Chest tube duration	15	− 0.63 [− 1.03, − 0.23]	0.002	84%	< 0.01
Blood loss	14	− 11.64 [− 26.34, 3.06]	0.12	97%	< 0.01
V.A.S	7	− 0.57 [− 0.97, − 0.18]	0.004	97%	< 0.01
L.O.S	17	− 0.54 [− 0.94, − 0.13]	0.009	87%	< 0.01
Resected lymph nodes	15	0.15 [− 0.72, 1.02]	0.74	73%	< 0.01
Median survival	3	1.22 [− 0.55, 2.99]	0.18	8%	0.34

*M.O.T.* mean operative time; *V.A.S.* visual analogy scale; *L.O.S.* length of stay; *OR* odds ratio; *WMD* weighted mean difference; *CI* confidence intervals

### Perioperative Parameters and Outcomes

Patients in both groups presented similar baseline respiratory function, as expressed by FEV1% (forced expiratory volume) [WMD: 0.11 (95% CI − 0.03, 0.25); *p* = 0.12]. No difference was reported between the two groups regarding the mean operative time (M.O.T.) [WMD: 9.37 (95% CI − 0.66, 19.40); *p* = 0.07], the mean intraoperative blood loss [WMD: − 11.64 (95% CI − 26.34, 3.06); *p* = 0.12], and the conversion rate [OR: 0.91 (95% CI 0.56, 1.46); *p* = 0.68]. Nonetheless, UVATS was associated with shorter chest tube duration [WMD: − 0.63 (95% CI − 1.03, − 0.23); *p* = 0.002] and length of stay (L.O.S.) [WMD: − 0.54 (95% CI − 0.94, − 0.13); *p* = 0.009] (Fig. 2). Pain was assessed using the visual analog score (V.A.S.) and was significantly lower in the UVATS group [WMD: − 0.57 (95% CI − 0.97, − 0.18); *p* = 0.004].

### Complications and Short-Term Mortality

The incidence of total complications was higher in patients undergoing MVATS [OR: 0.76 (95% CI 0.62, 0.93); *p* = 0.008] (Fig. 3). According to the subgroup analysis, the rate of postoperative arrhythmias and respiratory complications was similar between the two groups. No significant difference was demonstrated between UVATS and MVATS regarding the 30-day mortality [OR: 0.51 (95% CI 0.10, 2.47); *p* = 0.40].

### Oncologic Outcome and Long-Term Survival

Both UVATS and MVATS presented similar outcomes in terms of number of resected lymph nodes [OR: 0.15 (95% CI − 0.72, 1.02); *p* = 0.74]. *Only* three studies [10, 17, 30] provided outcomes on long-term survival. In fact, no significant difference was reported between the two groups regarding the survival at 1 year [OR: 0.80 (95% CI 0.24, 2.68); *p* = 0.72] and 3 years [OR: 0.73 (95% CI 0.25, 2.11); *p* = 0.56] postoperatively.

### Publication Bias

Heterogeneity was high regarding most of the outcomes. Nonetheless, heterogeneity was low regarding, the mortality and survival outcomes, along with the incidence of conversions and complications. The funnel plots that were produced to assess publication bias are shown in *Figure S2.* The asymmetries that were found are mainly attributed to the selection of the patients, along with the differences in technique and instruments among centers, thus, proposing that more well-designed studies are necessary to eliminate publication bias.

## Discussion

The current evidence regarding the benefits or even noninferiority of UVATS over MVATS for non-small cell lung cancer is limited, while there are only two RCT available with a small number of participants and short follow-up. In this context, the present study represents the highest level of evidence. Although a previous meta-analysis [31], published in 2017, compared to UVATS and MVATS, it included only eleven studies, since a significant number of studies were published between 2017 and 2020. Another study [32] demonstrated the superiority of UVATS over MVATS regarding perioperative outcomes, it included only 8 studies and a small patient population. In addition, a meta-analysis by Abouarab et al. [33] included various types of procedures, thus, posing a certain selection bias. The present meta-analysis included 20 articles describing UVATS and MVATS as alternative procedures for patients with non-small cell lung cancer, measuring patients’ perioperative, oncologic, and survival outcomes and published between 2014 and 2020.

Currently, no consensus has been reached regarding the superiority of either procedure, while the consensus report by UVIG-ESTS, published in 2019, was calling for newer evidence on the topic. The present study demonstrated that both procedures are relatively safe, with similarly low 30-day mortality rates. Nonetheless, UVATS presented lower total complication rate and shorter chest tube duration, thus, being associated with a higher level of safety. These outcomes are in accordance with the previous meta-analysis [31] and have a direct impact on clinical practice. In addition, patients undergoing UVATS presented significantly shorter L.O.S., probably due to the fewer complications and the shorter chest tube duration. Due to its minimally invasive nature, UVATS implements less traumatic manipulations, thus, reducing the intercostal nerve disorder, along with the postoperative pain [17]. As a result, patients present a lower complication rate and a faster recovery. We also analyzed specific complications, such as arrhythmias and respiratory complications, but without any significant difference being reported, possibly because the sample size was small. Furthermore, both procedures demonstrated similar M.O.T., conversion rate, and blood loss, thus being similarly feasible. Both M.O.T. and blood loss were associated with high heterogeneity. The main explanation includes the impact of the learning curve on both variables, along with the different instruments being employed by different institutions.

The number of retrieved lymph nodes was similar between the two groups. This finding is in accordance with the previous meta-analysis and suggests that the lymph node dissection performed by UVATS meets the oncologic requirements. This finding was further certified by our outcomes regarding the survival at 1 year and 3 years postoperatively, although more studies are necessary to fully elucidate the long-term survival outcomes. The high heterogeneity regarding the number of harvested lymph nodes is mainly attributed to the different strategies implemented among centers regarding the extent of lymph node dissection.

Given the small number of RCTs comparing the feasibility of UVATS and MVATS for non-small cell lung cancer, the current work is the largest up-to-date comparative study, implementing the UVIG-ESTS criteria, incorporating 1,469 patients treated with UVATS and 3,231 treated with MVATS. One meta-analysis [31] has been previously published, but included only eleven studies, thus limiting the value of the study. The present meta-analysis supports the outcomes of the previous while providing greater clarity regarding significant long-term survival endpoints. Given the enhanced perioperative outcomes of UVATS, we recommend the implementation of UVATS for patients with non-small cell lung cancer. However, the decision regarding the procedure of choice should be made on the basis of disease status, the institutional and surgeon experience, along with the patient’s opinion on the basis of a shared decision-making process.

This meta-analysis demonstrates the need for additional studies comparing UVATS and MVATS regarding the long-term survival and oncologic outcomes. Ideally, these would be multi-institutional randomized controlled studies, with a prospective design, well-specified inclusion, and exclusion criteria, clinical matching of the UVATS and MVATS groups, along with longer follow-up. These studies should also uncover whether special patient subgroups would better fit in the UVATS approach.

The present meta-analysis presents certain limitations that are associated with the included studies. The majority of the studies were retrospective, three studies were prospective, and there were only two RCTs, thus posing a certain bias in this study. In addition, the inter-institutional differences regarding the selection criteria for either UVATS or MVATS, along with the disparities regarding the perioperative management pose another limitation. In fact, the selection criteria were heterogeneous, and they have been, potentially, based on the patients’ clinical characteristics and status, thus posing a certain selection bias that could not been adjusted in the present study. Finally, the suboptimal coding regarding several variables, such as histology, may have affected the integrity of propensity matching, along with the survival outcomes. Furthermore, the pooled estimates of M.O.T., L.O.S., intraoperative blood loss, number of dissected lymph nodes, and chest tube duration are significantly heterogeneous, thus, indicating that certain factors associated with the basic surgical approach, the surgeon’s level of expertise, or the standardization of data definition across the different institutions, may have implicated. Finally, another limitation is the differences among institutions regarding the multimodal treatment protocols that have been applied to the included patients.

On the other hand, there are certain strengths in the present study: (1) a clear data extraction protocol, (2) well-specified inclusion–exclusion criteria, (3) the literature search was performed in three different databases, (4) a quality assessment of the included studies was performed, and (5) there was a detailed presentation of the results of data extraction and analysis.

## Conclusion

This meta-analysis identified 20 unique peer-reviewed studies comparing UVATS, and MVATS as alternative surgical options for patients with non-small cell lung cancer. These studies suggest that UVATS is associated with shorter chest tube duration, fewer complications, and shorter L.O.S. In this context, we recommend the implementation of UVATS as the procedure of choice for patients with non-small cell lung cancer. Nonetheless, the decision regarding the procedure of choice should be made on the basis of disease status along with the institutional/surgeon experience and the patient’s special interest. These results should be interpreted with caution due to the small number of RCTs. Future RCTs with greater clarity regarding significant outcomes, such as long-term survival and complications, are necessary in order to demonstrate the differences in efficacy between UVATS and MVATS.

## Supplementary Information

Below is the link to the electronic supplementary material.

Supplementary file1 (DOCX 25 KB)

Supplementary file2 (DOCX 28 KB)

Supplementary file3 (DOCX 37 KB)
