# Supplementary material for: Uniportal Versus Multiportal Video-Assisted Thoracoscopic Lobectomy for Lung Cancer: An Updated Meta-analysis
Source: Lung. 2021 Jan 2;199(1):43–53. doi: 10.1007/s00408-020-00411-9 (PMC7929953; doi:10.1007/s00408-020-00411-9)
Supplement: Supplementary file 1 — Supplementary file1 (DOCX 25 KB) [file 408_2020_411_MOESM1_ESM.docx]

| ***PICO*** | |
| --- | --- |
| P: Population of interest | Patients with lung cancer undergoing lobectomy |
| I: Intervention | Uniportal Video-assisted thoracoscopic surgery |
| C: Control | Conventional multiport video-assisted thoracoscopic surgery |
| O: Outcome | The perioperative short-term outcomes were the primary endpoints and the long-term survival was the secondary endpoint. |
