# Supplementary material for: Uniportal Versus Multiportal Video-Assisted Thoracoscopic Lobectomy for Lung Cancer: An Updated Meta-analysis
Source: Lung. 2021 Jan 2;199(1):43–53. doi: 10.1007/s00408-020-00411-9 (PMC7929953; doi:10.1007/s00408-020-00411-9)
Supplement: Supplementary file 2 — Supplementary file2 (DOCX 28 KB) [file 408_2020_411_MOESM2_ESM.docx]

| **References** | **Random sequence generation** | **Allocation concealment** | **Blinding of participants and personnel** | **Blinding of outcome assessment** | **Incomplete outcome data** | **Selective reporting** |
| --- | --- | --- | --- | --- | --- | --- |
| Perna et al., 2016 [24] | Low risk | Low risk | Low risk | Low risk | Low risk | Low risk |
| Zhang et al., 2020 [29] | Low risk | Low risk | High risk | High risk | Low risk | Low risk |

The RCTs were assessed for their methodological quality with the tools that are used to evaluate the risk of bias according to the Cochrane Handbook for Systematic Reviews of Interventions
