# Supplementary material for: Uniportal Versus Multiportal Video-Assisted Thoracoscopic Lobectomy for Lung Cancer: An Updated Meta-analysis
Source: Lung. 2021 Jan 2;199(1):43–53. doi: 10.1007/s00408-020-00411-9 (PMC7929953; doi:10.1007/s00408-020-00411-9)
Supplement: Supplementary file 3 — Supplementary file3 (DOCX 37 KB) [file 408_2020_411_MOESM3_ESM.docx]

| **STUDY ID, YEAR** | **HISTOLOGY, n (%)** | | | | | | | | **STAGE, n** | | | | **Tumor size, cm, mean (SD)** | |
| --- | --- | --- | --- | --- | --- | --- | --- | --- | --- | --- | --- | --- | --- | --- |
|  | **Squamous** | | **Adenocarcinoma** | | **Carcinoid** | | **Other** | | **I/II** | | **III/IV** | |  |  |
|  | **UVATS** | **MVATS** | **UVATS** | **MVATS** | **UVATS** | **MVATS** | **UVATS** | **MVATS** | **UVATS** | **MVATS** | **UVATS** | **MVATS** | **UVATS** | **MVATS** |
| Al-Ameri et al. 2019 | 11  (9.0) | 18  (8.5) | 95  (77.9) | 162  (76.8) | 10  (8.2) | 19  (9.0) | 6  (4.9) | 12  (5.7) | 112  (91.8) | 201  (95.2) | 10  (8.2) | 10  (4.7) | N/A | N/A |
| Bin Yameen et al. 2019 | 5  (7.7) | 8  (12.7) | 48  (73.8) | 36  (57.1) | 2  (3.1) | 3  (4.8) | 3  (4.6) | 5  (7.9) | 52  (80.0) | 44  (69.8) | 7  (10.7) | 8  (12.7) | 2.7  (1.5) | 2.8  (1.7) |
| Bourdages –Pageau et al. 2019 | 41  (17) | 36  (15) | 185  (75) | 188  (76) | 0 | 0 | 21  (9) | 23  (9) | 221  (89.4) | 226  (91.4) | 26  (10.5) | 21  (8.5) | 2.5  (1.2) | 2.4  (1.2) |
| Chung et al. 2015 | 15  (16.7) | 8  (13.3) | 55  (61.1) | 45  (75) | 0 | 0 | 7  (7.8) | 2  (3.3) | 63  (70) | 44  (73.3) | 27  (30) | 14  (23.3) | N/A | N/A |
| Dai et al. 2016 | 9  (14.29) | 12  (19.05) | 54  (85.71) | 51  (80.95) | 0 | 0 | 0 | 0 | 46  (73) | 44  (69.8) | 17  (26.9) | 19  (30.1) | 3.92  (1.57) | 4.21  (1.66) |
| Han et al. 2016 | N/A | N/A | N/A | N/A | N/A | N/A | N/A | N/A | N/A | N/A | N/A | N/A | N/A | N/A |
| Heo et al. 2017 | 7  (21.9) | 7  (21.9) | 23  (7.9) | 23  (7.9) | 0 | 0 | 2  (6.3) | 2  (6.3) | 28  (87.5) | 30  (93.7) | 4  (12.5) | 2  (6.3) | 2.5  (1.3-6.0) | 2.5  (1.0-5.5) |
| Hirai et al. 2015 | N/A | N/A | N/A | N/A | N/A | N/A | N/A | N/A | N/A | N/A | N/A | N/A | 2.4  (1.1-3.5) | 1.8  (0.8-3.2) |
| Hirai et al. 2015 | N/A | N/A | N/A | N/A | N/A | N/A | N/A | N/A | N/A | N/A | N/A | N/A | N/A | N/A |
| Jiménez et al. 2017 | N/A | N/A | N/A | N/A | N/A | N/A | N/A | N/A | N/A | N/A | N/A | N/A | N/A | N/A |
| Ke et al. 2017 | 3  (7.5) | 6  (15.0) | 37  (92.5) | 34  (85.0) | 0 | 0 | 0 | 0 | 40  (100) | 40  (100) | 0 | 0 | 1  (<1-3) | 1-2  (<1-3) |
| Kim et al. 2017 | N/A | N/A | N/A | N/A | N/A | N/A | N/A | N/A | N/A | N/A | N/A | N/A | N/A | N/A |
| Lin et al. 2016 | N/A | N/A | N/A | N/A | N/A | N/A | N/A | N/A | N/A | N/A | N/A | N/A | N/A | N/A |
| McElnay et al. 2014 | N/A | N/A | N/A | N/A | N/A | N/A | N/A | N/A | N/A | N/A | N/A | N/A | N/A | N/A |
| Mu et al. 2015 | 2  (4.3) | 5  (10.6) | 45  (95.7) | 42  (89.4) | 0 | 0 | 0 | 0 | 36  (76.5) | 31  (65.9) | 2  (4.3) | 1  (2.1) | 2.02  (1.30) | 1.62  (1.04) |
| Perna et al. 2016 | 16  (31.4) | 17  (30.9) | 35  (68.6) | 38  (69.1) | 0 | 0 | 0 | 0 | 35  (68.6) | 40  (72.7) | 16  (31.3) | 15  (27.3) | N/A | N/A |
| Shen et al. 2015 | 20  (20) | 16  (16) | 80  (80) | 84  (84) | 0 | 0 | 0 | 0 | 100  (100) | 100  (100) | 0 | 0 | 2.53  (0.86) | 2.50  (0.90) |
| Song et al. 2017 | 8  (30.8) | 7  (26.9) | 17  (65.4) | 19  (73.1) | 0 | 0 | 1  (3.8) | 0 | 26  (100) | 25  (96.1) | 0 | 1  (3.8) | 2.32  (1.48) | 2.32  (0.9) |
| Tosi et al. 2019 | 19  (11.0) | 228  (12.6) | 128  (74.5) | 1330  (73.6) | N/A | N/A | 25  (14.5) | 250  (13.8) | N/A | N/A | N/A | N/A | 2  (<2-7) | 2  (<2- >7) |
| Xu et al. 2019 | 10  (16.6) | 8  (13.3) | 47  (78.3) | 48  (80) | N/A | N/A | 3  (5.0) | 4  (6.6) | 49  (81.6) | 50  (83.3) | 11  (18.3) | 10  (16.6) | N/A | N/A |
| Zhang et al. 2020 | N/A | N/A | N/A | N/A | N/A | N/A | N/A | N/A | N/A | N/A | N/A | N/A | N/A | N/A |
| Zhao et al. 2019 | 27  (37.0) | 16  (28.6) | 46  (63.0) | 40  (71.4) | 0 | 0 | 0 | 0 | 73  (100) | 56  (100) | 0 | 0 | 2.9  (0.8) | 2.9  (0.9) |
